# Precision mapping of mandibular canal dimensions: cone beam computed tomography insights

**DOI:** 10.7717/peerj.20553

**Published:** 2026-01-13

**Authors:** Mohamed Omar Elboraey, Emad El Said Fahim Essa, Mostafa Ibrahim Fayad, Albraa Badr Alolayan, Ahmed Mohamed Kabli, Abdullah Alqhtani, Nahla Gamaleldin Elhelbawy, Tarek Mohamed Ibrahim

**Affiliations:** 1 College of Dentistry, Taibah University, Al Madinah Almunawwarah, Saudi Arabia; 2 Oral Medicine, Periodontology, Oral Diagnosis and Oral Radiology Department, Faculty of Dentistry, Tanta University, Tanta, Egypt; 3 Faculty of Dentistry, Tanta University, Tanta, Egypt; 4 Faculty of Dental Medicine, Al Azhar University, Cairo, Egypt

**Keywords:** Cone beam computed tomography, Inferior alveolar canal, Mandibular anatomy, Mental foramen, Buccal cortex thickness

## Abstract

**Background:**

The purpose of this study was to provide detailed anatomical localization of the inferior alveolar canal (IAC) in the mandible area using cone beam computed tomography (CBCT) in the Saudi population, with particular considerations including buccal cortex thickness (BCT), IAC diameter, mental foramen position (MF), and buccal shift patterns of the IAC.

**Methods:**

This retrospective split-mouth study analyzed 126 CBCT scans of 63 males and 63 females between the age group of 18 to 65 years from the faculty of Dentistry, Taibah University, Saudi Arabia. Measurements included BCT at nine points between the first premolar and third molar area, IAC diameter, the closest point of IAC to the mandibular lower border, MF position in relation to premolar apices and mandibular border, and the location of buccal shifting of the IAC. Statistics were calculated with Student’s *t*-test and paired *t*-test with the level of significance at *p* ≤ 0.05.

**Results:**

BCT showed a progressive increase from anterior to posterior regions in both genders, with the thickest measurements at the second and third molar regions (maximum: 6.05 ± 1.34 mm in females). The MF position showed considerable individual variation, with 10% of female subjects exhibiting a coronal position relative to premolar apices. The IAC diameter ranged from 2.43 to 3.80 mm. The closest position of the IAC to the lower border was mostly in the second molar area, with the shortest distances being 2.50–3.40 mm. The canal primarily exhibited a buccal shift in the second molar region in 60–81.8% of the subjects.

**Conclusion:**

This study provides comprehensive anatomical mapping of the mandibular region, highlighting considerable individual variation in key anatomical parameters. The findings have significant clinical implications for various dental and maxillofacial procedures.

## Introduction

The mandible, the strongest facial bone, houses the inferior alveolar nerve (IAN) and vessels within the mandibular canal. An understanding of the course, dimensions, and anatomical relations of the inferior alveolar canal (IAC) is critical for clinical applications like implant placement, third molar extractions, orthognathic surgery, trauma management, and endodontic treatments (Juodzbalys et al., 2013; Ghaeminia et al., 2011).

The IAC shows various anatomical variations, increasing the risk of neurovascular complications during surgical procedures, ranging from paresthesia to complete anesthesia of the lower lip and chin (Naitoh et al., 2009; Bartling, Freeman & Kraut, 1999). During third molar extractions, IAN injury rates range from 0.4% to 8.4% (Jerjes et al., 2010).

The mandibular buccal cortex thickness (BCT) is another critical anatomical landmark with considerable variation, impacting dental implant selection, placement protocols, and necessity for buccal augmentation, through direct effects on implant stability and fenestration or dehiscence risk (Cassetta et al., 2013; Lee et al., 2021).

IAC terminates anteriorly at mental foramen (MF), accurate localization of the MF is critical for different surgical procedures as implant placement, periapical surgery, and autogenous bone graft harvesting from the chin (Al-Mahalawy et al., 2017; Greenstein & Tarnow, 2006).

Cone beam computed tomography (CBCT) provides 3D imaging of maxillofacial structures with easy interpretation and layering of scans. It also provides a lower radiation dose than computed tomography (CT) scans and outperforms 2D panoramic radiographs, which provide limited 2D information for 3D structures (Angelopoulos et al., 2008; Scarfe, Farman & Sukovic, 2006).

CBCT interpretation of IAC provides more accurate preoperative planning and risk assessment, reduces neurovascular complications during invasive surgical procedures by 90% (Kamburoğlu et al., 2015; Ghaeminia et al., 2011).

However, despite the clinical significance, studies for comprehensive IAC mapping are limited especially in Middle Eastern populations. However, comprehensive 3D mapping studies of the IAC, including BCT, IAC diameter, and MF position using CBCT are still limited, especially within Middle Eastern populations. Existing research often focuses on specific anatomical features or uses less detailed 2D imaging (Muñoz et al., 2017; Ozturk, Potluri & Vieira, 2012)

In addition, gender and side-based mandibular anatomical variations remain underexplored, despite their clinical relevance. Identifying such variations could inform gender or side-specific surgical planning and approaches (Kamburoğlu et al., 2013).

This study aimed at a comprehensive mapping of the IAC using CBCT to assess gender and side-based mandibular anatomical variations, having direct clinical implications to various dental and maxillofacial procedures, to enhanced preoperative planning and reduced neurovascular complications risk.

## Materials & Methods

This split-mouth study was conducted retrospectively for mapping the IAC of the mandible using CBCT scans from the College of Dentistry, Taibah University, KSA. Ethical approval was obtained from the ethics committee (TUCDREC/231024/Eessa). Informed consent was waived by the Research Ethics Committee at Taibah University, College of Dentistry (TUCD-REC).

A total 126 CBCT scans from 63 male and 63 female subjects were involved in the study, with an age range of 18 to 65 years. The used sample size formula was *n* = ((*Zα*/2⋅*σ*)/*E*)^2^. Where *Zα*/2 is the critical value for 95% confidence, *σ* is the standard deviation, and E is the margin of error. The anticipated Standard Deviation (*σ*) was conservatively estimated at 1.5 mm based on published data for mandibular morphometric measurements in similar CBCT studies (Kamburoğlu et al., 2009). With a 95% confidence level (Z*α*/2 = 1.96) and a desired margin of error (E) of 0.5 mm. The minimum sample size was 35 per group, increased to 63 to improve statistical power.

### Inclusion and exclusion criteria

#### Inclusion criteria

(1) CBCT scans of adult patients (≥18 years)

(2) patients with fully erupted mandibular posterior dentition.

#### Exclusion criteria

(1) Scans exhibiting with significant artifacts affecting the measurements.

(2) History of mandibular trauma or surgery.

(3) Presence of mandibular pathological lesions and/or developmental anomalies.

### Image acquisition and analysis

All CBCT scans were analyzed using Carestream 3D Imaging Software (Carestream Dental LLC, Atlanta, GA, USA). All CBCT scans were acquired following the standardized imaging protocol of the Taibah University Dental Clinic, which mandates patient positioning in the Frankfort Horizontal plane prior to image acquisition to ensure reproducible head orientation. Scans were acquired at 90 kVp, 14.55 mAs, 160 µm voxel size, and 15.5 s. Images were reconstructed at 0.1 mm slice thickness and measurements were taken to 0.01 mm precision using digital calipers.

All measurements were performed by two calibrated examiners. To ensure reliability, 20% of the scans were re-measured after two weeks to calculate intra- and inter-examiner reliability. All continuous linear measurements were assessed using the Intraclass Correlation Coefficient (ICC). The intra-examiner ICC was 0.95 and the inter-examiner ICC was 0.92, indicating excellent agreement for all parameters measured in the study.

### Measurement parameters

#### Buccal cortex thickness (BCT)

**Figure 1:**
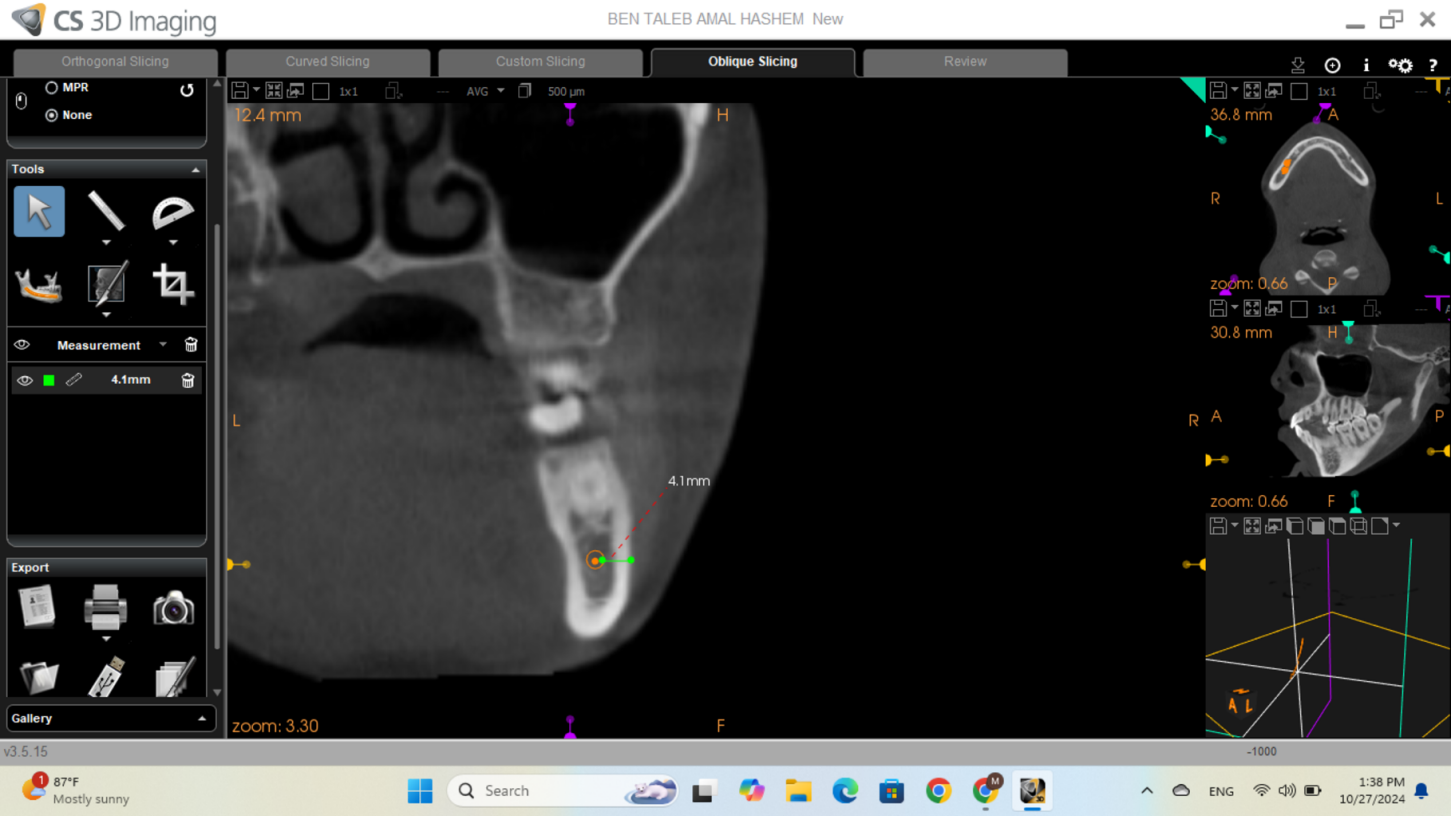
Cross-sectional view of CBCT for buccal cortex thickness measurement.

BCT was measured at nine sites from the first premolar (tooth #4) to the third molar (tooth #8), including interdental regions, using CBCT cross-sectional views from the buccal cortex of the IAC to the most buccal point of the mandibular buccal cortex at the specified locations as shown in Fig. 1.

#### Buccal shifting location

The point of buccal shifting of the IAC was identified using panoramic, then monitored in axial CBCT views until the buccal shift was clearly observed and recorded relative to adjacent teeth.

#### Location of the mental foramen (MF)

The location of the MF was identified and measured from the MF center to three different points in millimeters using the sagittal view of CBCT: the apices of the first premolar and second premolar, and the lower border of the mandible.

#### Diameter of the inferior alveolar canal

**Figure 2:**
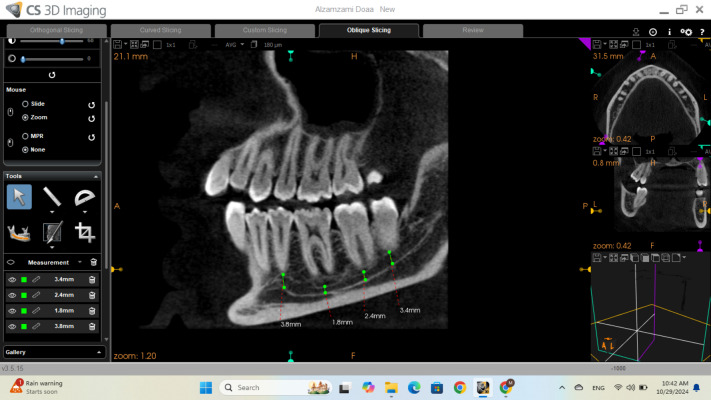
Sagittal view of CBCT showing the measurement of the inferior alveolar diameter.

The diameter of the IAC was measured at five locations next to the apices of first and second premolars and the first, second, and third molar teeth using the cross-sectional view from CBCT, then refined on the sagittal view to ensure accurate determination of the canal diameter as shown in Fig. 2.

#### Closest point from the inferior alveolar canal to the lower border of the mandible

Using the sagittal view of CBCT, the distance between the lower borders of the IAC and the mandible was measured at multiple points along the canal. The minimum distance was recorded, and its location was noted in relation to the corresponding tooth.

### Statistical analysis

Data was analyzed using IBM SPSS Statistics v20.0 (IBM Corp., Armonk, NY, USA). Categorical data were expressed as numbers and percentages; comparisons used the Chi-square test, with Fisher’s Exact or Monte Carlo corrections when >20% of cells had expected counts <5. Normality of continuous data was assessed using the Shapiro–Wilk test. Quantitative data were summarized as range, mean, and SD. Student’s *t*-test was used for comparing two independent groups, and the paired *t*-test for related samples. Significance was set at *p* ≤ 0.05.

## Results

This retrospective study analyzed 126 CBCT scans to map the IAC, various anatomical landmarks were measured bilaterally including BCT, IAC diameter, proximity of the IAC to the mandibular border, MF position, and buccal shifting location of IAC.

### Buccal cortex thickness

The BCT was measured at nine locations bilaterally, from the first premolar to the third molar region with no statistically significant differences between male and female or right and left sides (*p* > 0.05). However, notable anatomical patterns were identified.

**Figure 3:**
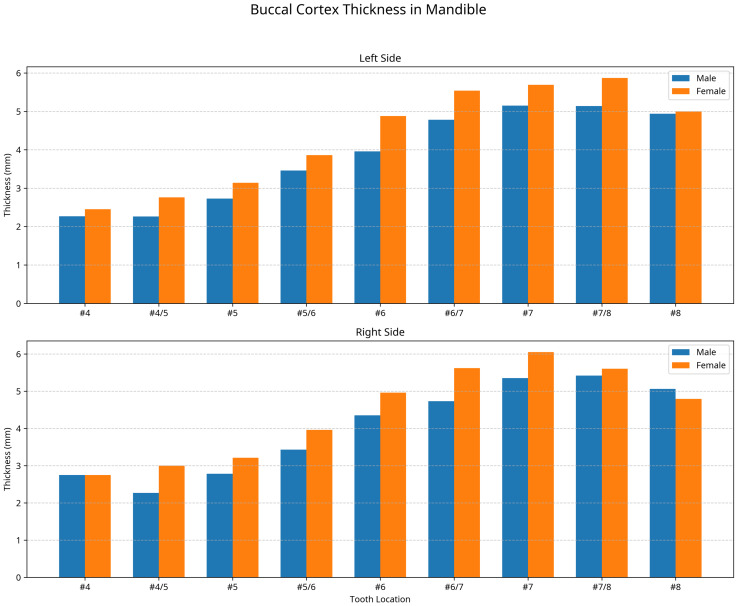
Buccal cortex thickness in (mm) at the left and right side for males and females.

For female subjects, the BCT was generally thicker than in male subjects, showing a maximum difference of 0.92 mm on the left side and 0.89 mm on the right side. The maximum recorded value for BCT in females was at tooth 47 by 6.05 ± 1.34 mm, while in males was measured at the midpoint between teeth 37 and 38 by 5.14 ± 1.24 mm and at the midpoint between teeth 47 and 48 by 5.42 ± 1.58 mm as shown in Fig. 3.

At the premolar region (tooth 34), the mean BCT was 2.27 ±  0.95 mm in males and 2.45 ± 1.20 mm in females. Measurements at this location were only possible in 27.27% of males and 20% of females, representing the anatomical looping of the nerve at the left side. Similarly, at tooth 44, measurements were possible in 18% of males and 40% of females as shown in Fig. 3.

Table 1 showed the detailed measurements of BCT at all locations for both genders and sides. The data shows a consistent pattern of gradual increase in thickness from the premolar to the molar region, with the thickest measurements recorded in the second and third molar regions.

**Table 1:** Comparison between the two studied groups according to mental foramen position.

	Mental foramen position	Left	Right	t	*p*
Male	**Apex of #4**				
	Min.–Max.	4.60–12	4.80–13.10	0.346	0.737
	Mean ± SD.	7.78 ± 2.40	7.98 ± 2.22		
	**Apex of #5**				
	Min.–Max.	2.80–10.10	2.50–11.60	1.184	0.264
	Mean ± SD.	6.64 ± 2.45	7.51 ± 2.70		
	**Lower border**				
	Min.–Max.	8.50–14.70	7.50–13.10	2.905	0.16
	Mean ± SD.	11.51 ± 1.83	10.71 ± 1.99		
Female	**Apex of #4**				
	Min.–Max.	3.20–11.30	−3.30–9.80	1.895	0.091
	Mean ± SD.	7.42 ± 2.27	5.98 ± 3.94		
	**Apex of #5**				
	Min.–Max.	−2.50–8.50	−4.70–10.40	0.141	0.891
	Mean ± SD.	6.11 ± 3.30	6.03 ± 4.25		
	**Lower border**				
	Min.–Max.	7.90–12.70	7.80–13.30	0.795	0.447
	Mean ± SD.	10.05 ± 1.62	10.30 ± 1.86		

**Notes.**

SD Standard deviation t Paired *t*-test p *p* value for comparing between Left and Right * Statistically significant at *p* ≤ 0.05

### Mental Foramen location

The position of the MF was assessed in relation to the apices of the first and second premolars and the lower border of the mandible. No statistically significant differences were found between sides or genders (*p* > 0.05) as shown in Table 1.

The mean distance from the mental foramen (MF) to the lower border of the mandible is greater in male than female subjects at left and right side by 1.45 and 0.45 mm respectively. While the mean distance from the mental foramen (MF) to the apex of the first premolar was generally greater than that to the second premolar, except on the right side, which exhibited an inverse pattern. Females demonstrated significantly greater variability in MF–premolar distances, particularly on the right side (SD = 3.94 mm and 4.25 mm). Notably, the mean MF–first premolar distance on the right side was significantly smaller in females (5.98 ± 3.94 mm) compared to males (7.98 ± 4.25 mm; *p* < 0.05), indicating a pronounced sex-related difference in the anteroposterior positioning of the MF.

An important observation was the presence of negative values in 10% of female subjects bilaterally, indicating a coronal position of the MF relative to the premolar apices. This anatomical variation was not observed in male subjects.

### Diameter of the inferior alveolar canal

The diameter of the IAC was measured at five locations corresponding to teeth #4 through #8. No significant differences were found between genders or sides (*p* > 0.05) as shown in Table 2.

**Table 2:** Comparison between the two groups studied according to the canal diameters related to each tooth.

	Canal diameters related to each tooth	Left	Right	t	*p*
Male	**4**				
	Min.–Max.	0.80–4.40	2.20–2.90	0.753	0.325
	Mean ± SD.	2.43 ± 1.82	2.47 ± 0.38		
	**5**				
	Min.–Max.	1.70–4.90	2.70–5.30	0.844	0.419
	Mean ± SD.	3.40 ± 1.20	3.71 ± 0.78		
	**6**				
	Min.–Max.	2.30–4.10	2.50–4.50	0.198	0.847
	Mean ± SD.	3.19 ± 0.56	3.25 ± 0.70		
	**7**				
	Min.–Max.	2.50–5	2.70–4.10	0.611	0.555
	Mean ± SD.	3.57 ± 0.79	3.42 ± 0.43		
	**8**				
	Min.–Max.	2.40–4.90	2.10–4.30	1.416	0.190
	Mean ± SD.	3.80 ± 0.73	3.29 ± 0.65		
Female	**4**				
	Min.–Max.	2.10–3.10	1.40–2.90	0.600	0.656
	Mean ± SD.	2.60 ± 0.71	2.45 ± 0.71		
	**5**				
	Min.–Max.	2.30–4.30	2.10–8.30	0.812	0.438
	Mean ± SD.	3.41 ± 0.66	3.78 ± 1.74		
	**6**				
	Min.–Max.	1.80–4.70	2.50–7.40	1.070	0.312
	Mean ± SD.	2.93 ± 0.77	3.50 ± 1.45		
	**7**				
	Min.–Max.	2.20–4.50	2.10–3.80	0.718	0.491
	Mean ± SD.	3.13 ± 0.63	2.97 ± 0.63		
	**8**				
	Min.–Max.	2.50–3.90	2.10–3.80	1.036	0.327
	Mean ± SD.	3.10 ± 0.44	2.88 ± 0.51		

**Notes.**

SD Standard deviation t Paired *t*-test *p* *p* value for comparing between Left and Right

* Statistically significant at *p* ≤ 0.05.

In male subjects, the canal diameter ranged from 2.43 ±  1.82 mm (at tooth 34) to 3.80 ± 0.73 mm (at tooth 38) on the left side, and from 2.47 ± 0.38 mm (at tooth 44) to 3.71 ±  0.78 mm (at tooth 45) on the right side.

In female subjects, the canal diameter ranged from 2.60 ±  0.71 mm (at tooth 34) to 3.41 ± 0.66 mm (at tooth 35) on the left side, and from 2.45 ± 0.71 mm (at tooth 44) to 3.78 ±  1.74 mm (at tooth 45) on the right side.

Variability in IAC diameter was assessed by coefficient of variation (CV), it was highest in premolar regions. In males, CV ranged from 12.6% (tooth 47) to 35.3% (tooth 35). In females, it ranged from 14.2% (tooth 38) to 46% (tooth 45), indicating significant anatomical variation.

### Closest point of inferior alveolar canal to the lower border of the mandible

The minimum distance between the IAC and the lower border of the mandible was measured. Although not statistically significant, the canal was generally closer to the lower border in males than in females, and on the right side compared to the left side.

In male subjects, the minimum distances were 4.56 ± 1.50 mm on the left side at teeth 36 and 37 and 4.85 ± 1.68 mm on the right side at tooth 47.

In female subjects, the minimum distances were 4.96 ± 1.08 mm on the left side at tooth 37 and 5.09 ± 0.96 mm on the right side at tooth 48.

The IAC was closest to the lower border most frequently in the second molar region. In females, this occurred at tooth 47 (60%) and 37 (40%) bilaterally. Among males, the right side showed the highest frequency (72.7%), followed by the left (45.5%).

### Location of the buccal shifting of inferior alveolar canal

Buccal shifting of the IAC was most frequent at the first and second molar regions in both genders. In males, it occurred at second molar tooth by 81.8% and 72.7% of cases on the right and the left sides respectively. In females, it was seen at the second molar in 70% of cases on the right and 60% on the left.

These findings provide detailed anatomical mapping of the IAC and associated structures, highlighting important gender-based and side-based variations that have clinical implications for dental and maxillofacial procedures in the posterior mandibular region.

## Discussion

This retrospective CBCT study provides an anatomical mapping of the IAC, BCT, and MF in Saudi population, contributing relevant insights for oral and maxillofacial surgery, implantology, and related dental practices.

### Buccal cortex thickness

A progressive increase in BCT from premolar to molar was noted, consistent with Kawashima et al. (2016). Females exhibited slightly thicker BCT than males, though the difference was not statistically significant, contrasting with studies like (Cassetta et al., 2013) and (Kamburoğlu et al., 2013). Possibly due to ethnic variability in mandibular anatomy as observed by Chung et al. (1995).

Nerve looping at tooth 34 (27.27% males and 20% females) underscores the anatomical complexity and risk zones, aliening with (Naitoh et al., 2009; Khorshidi et al., 2017), emphasizing the necessity for preoperative CBCT.

Clinically, a BCT less than 2 mm increases IAN injury risk (Park et al., 2010). Our minimum BCT over third molar was 2.96 mm, suggesting lower risk for the Saudi population.

For internal fixation, ≥2.5 mm of BT is required for 2.0 mm screws (Tank, 2016). Mean BCT at tooth 34 was 2.27 ± 0.95 mm (males) and 2.45 ±  1.20 mm (females); measurements at tooth 44 showed similar values. Additionally, canal diameter ≥ 3.8 mm has been associated with increased risk of IAN injury, the values were suboptimal in most males and some females, supporting the use of shorter screws (2.5–3.0 mm) or supplementary fixation is clinically advisable.

For the molar region, BCT ≥4 mm allows safe four mm screw fixation. Our measurements (females: 6.05 mm; males: 5.42 mm) exceed this threshold, indicating safe surgical zones. Basal implant stability requires 2.0−2.5 mm BCT with 3–5 mm being optimal (Gil et al., 2022; Wadhwani et al., 2024). Molar values in our sample were within the optimal range, while premolars were near the minimum, necessitating narrow implants and torque control.

### Mental foramen position

MF position showed considerable variation in its position relative to premolar apices and the mandibular border, consistent with Greenstein & Tarnow (2006); Juodzbalys, Wang & Sabalys (2010). The mean distance to the lower mandibular border showed that the mandible size is greater in males than females subjects with a range from 10.05–11.51 mm, aligning with values reported by (Al-Mahalawy et al., 2017; Budhiraja et al., 2013).

A negative value was observed in 10% of females, indicating a coronal position of the MF relative to premolar apices, which was not seen in males. Similar variations have been reported by Kalender, Orhan & Aksoy (2012); Khojastepour et al. (2015), although at different frequencies, further emphasizing the importance of preoperative imaging assessment, especially for apical surgeries and implant placement in the premolar region.

Clinically, the measured MF–premolar distances (5.98–7.98 mm for the first premolar and 6.03–7.51 mm for the second) support using the second premolar as a reliable landmark point for mental nerve blocks, ensuring effective anaesthesia and low morbidity (Halwani & Muteq, 2018). When this distance is ≥5 mm, standard diameter implants can be safely placed 3.5–4.5 mm lingual to the socket, with the recommended 2 mm safety margin from the foramen (AlQahtani, 2022).

Fixation with 8–10 mm screws along the mandibular border is considered safe for general procedures in our study population, provided preoperative CBCT confirms ≥10 mm MF–border distance. This ensures screw tips >3.5 mm above the IAC, and 2.0–2.4 mm plates can be used without intraoperative imaging (Lee, Sawhney & Ducic, 2013).

Despite alveolar ridge resorption and reduced crest foramen height, the MF -basal border distance remains relatively stable, making it a reliable landmark for prosthetic planning. Shaping prosthesis flanges to maintain ≥ 2 mm clearance from the MF minimizes the potential for chronic nerve compression under functional movements, even in severely resorbed edentulous mandibles.

This CBCT-based mapping of the posterior mandible is in agreement with regional findings by Algabri et al. (2025) demonstrating high bilateral consistency in MF position, establishes essential reference parameters for safe surgical practice in the Saudi population. Although the MF generally shows bilateral symmetry, data revealed a distinct gender-related variation—a coronal position relative to the premolar apices in 10% of female subjects. This clinically relevant deviation highlights the need for individualized assessment rather than reliance on generalized anatomical norms. Therefore, preoperative CBCT evaluation should be regarded as a standard of care for all interventions in the mandibular premolar region, including implant placement and periapical surgery, to accurately define the MF’s three-dimensional position and prevent neurovascular complications.

### Diameter of the inferior alveolar canal

The IAC diameter varied significantly across different regions and individuals, with coefficients of variation ranging from 12.6% to 46%, underscoring the need for patient-specific assessment before invasive procedures. Our results align with Oliveira-Santos et al. (2011); Kamburoğlu et al. (2015), who also reported significant individual differences in canal dimensions.

The mean canal diameters in our study were 2.43−3.80 mm are comparable to those reported by Levine, Goddard & Dodson (2007) (2.0−3.4 mm) and Yashar et al. (2012). Slightly higher values may reflect differences in population or measurement technique. The observation of larger diameters in molar region aligns with findings by Gerlach et al. (2010); Hsu et al. (2013), who noted similar regional patterns.

Regarding the clinical significance of IAC diameter, normally ranges from 2 to 3 mm. In this study, the mean IAC diameter in the Saudi population ranged from 2.43 to 3.8 mm, falls within a relatively safe zone. Diameters >3.8 mm may increase the risk of IAN injury during implant placement, while those <2 mm raise the likelihood of canal penetration during osteotomy (Peña Cardelles et al., 2025). Diameters exceeding 4 mm may indicate early signs of perineural tumors or vascular malformations (Mortazavi et al., 2019).

Furthermore, current research necessitates the consideration that a smaller main IAC diameter may not merely reflect the nerve’s dimensions. Instead, it could serve as a critical indicator for the presence of accessory mandibular canals (AMCs) in the region, thereby substantially elevating the complexity of surgical intervention. Notably, Sözen & Akpınar (2025) reported that a reduced IAC diameter was significantly linked to the occurrence of AMCs, a finding observed in 36.9% of 222 hemimandibles examined.

### Proximity of the inferior alveolar canal to the inferior border of the mandible

IAC was typically closer to the mandibular lower border in males on the right side, though these differences were not statistically significant. The closest point was most frequently located in the second molar region, consistent with findings by Ozturk, Potluri & Vieira (2012).

The minimum distances recorded were 2.50−3.40 mm, slightly higher than those reported by Watanabe et al. (2010) with 1.8−2.2 mm but comparable to (Levine, Goddard & Dodson, 2007) with 2.5−3.3 mm. These variations may reflect population or measurement differences. Clinically, such distances are relevant to IAN injury risk during procedures like sagittal split osteotomy, open reduction with miniplates, and miniplate fixation for mandible fractures and mandibular reconstruction (Yamada et al., 2011; Juodzbalys et al., 2013).

### Location of the buccal shifting of inferior alveolar canal

Accurately identifying the buccal shift of the IAC is essential during posterior mandibular surgeries such as third molar extraction, implant placement, and sagittal split osteotomy. Buccal displacement increases IAN injury risk due to greater vulnerability during buccal access, as reported by Ghaeminia et al. (2009); Juodzbalys et al. (2013).

Buccal shifting of the IAC was most frequently observed in the first and second molar regions in both sexes, with the second molar being the most common site (60–81.8% of cases). This aligns with findings by Ozturk, Potluri & Vieira (2012); Naitoh et al. (2009).

### Clinical implications

Key clinical applications include:

Implant placement: Close IAC-buccal cortex relation in molar regions demands careful planning; increased posterior buccal thickness may improve implant stability.

Third molar surgery: Buccal displacement of the IAC at the second molar should guide bone removal to avoid nerve injury.

Sagittal split osteotomy: High variability in IAC–lower border distance, especially at the second molar, supports individualized CBCT-based planning.

Mental nerve procedures: Variability in mental foramen position, particularly in females, requires precise imaging before intervention.

Gender-based considerations: Trends such as thicker buccal cortex in females and lower IAC position in males suggest potential value in sex-specific evaluation.

Regarding their clinical significance, accessory canals may cause unexpected bleeding or neurosensory injury even when the main canal is avoided, highlighting the necessity of their evaluation using CBCT.

While our findings provide a precise anatomical map for the Saudi population, their clinical relevance is amplified when contextualized against established global morphometric norms, highlighting the significant role of ethnic variability in surgical planning. Our mean IAC diameter (ranging from 2.43 to 3.80 mm) falls on the larger end of the spectrum when compared to studies on East Asian populations, such as Taiwanese cohorts, where the mean diameter was reported at approximately 2.13 mm using similar CBCT methodology. This subtle increase in canal size necessitates even greater caution during osteotomy procedures. Conversely, the mean mental foramen-to-lower border distance in our study 10.05 to 11.51 mm) aligns closely with global averages, including those reported in Caucasian and American populations (9.84 to 10.52 mm), suggesting this vertical dimension is a more conserved evolutionary trait across diverse ethnic groups. However, the consistent pattern of a buccal shift of the IAC in the molar region (60–81.8%) and the specific coronal MF position in 10% of Saudi females represent distinct, clinically impactful variations that underscore the insufficiency of generalizing from non-Middle Eastern anatomical literature. These ethnic and gender-specific patterns mandate the establishment of regional safety parameters to reduce the risk of neurovascular injury in the local population (Chen et al., 2013).

### Limitations and future directions

This study has several limitations. First, the sample was limited to a single geographical area (Saudi Arabia). Second, certain anatomical measurements, such as bifid canals, accessory canals, accessory mental foramina, and canal-to-root proximity, were not taken and should be included in future studies for more complete anatomical mapping. Given the retrospective design and the use of a 160 µm voxel size, certain methodological limitations may have influenced the precision and consistency of the linear measurements. Although the ICCs demonstrated strong reliability, a minor degree of observer-related variability remains inevitable and may have contributed to slight discrepancies in measurement values. Finally, the creation of artificial intelligence algorithms for automatic detection and measurement of anatomical landmarks has the potential to improve the efficiency and accuracy of preoperative evaluations, a promising avenue for future research investigations.

## Conclusions

This CBCT-based assessment of the posterior mandible revealed several important findings. Marked individual variation was observed in the dimensions of the inferior alveolar canal (IAC) and the position of the mental foramen (MF), emphasizing the importance of individualized anatomical evaluation rather than reliance on generalized reference data. Additionally, significant sex-related differences were identified in (insert key parameter, *e.g.*, bone thickness or IAC diameter), suggesting that gender should be considered an important factor in surgical planning.

Clinically, the detailed analysis of buccal cortical thickness (BCT) and the spatial relationship between the IAC and MF underscores the value of preoperative CBCT imaging. Understanding these patient-specific anatomical features is crucial for minimizing the risk of nerve injury and enhancing safety and precision in procedures such as dental implant placement, mandibular third molar extraction, and sagittal split osteotomy.

##  Supplemental Information

10.7717/peerj.20553/supp-1 Supplemental Information 1 Raw data before statistical analysis

10.7717/peerj.20553/supp-2 Supplemental Information 2 STROBE checklist

## Additional Information and Declarations

### Data Availability

The following information was supplied regarding data availability: The raw data is available in the Supplemental Files.
