# Supplementary material for: Precision mapping of mandibular canal dimensions: cone beam computed tomography insights
Source: PeerJ. 2026 Jan 13;14:e20553. doi: 10.7717/peerj.20553 (PMC12810362; doi:10.7717/peerj.20553)
Supplement: Supplemental Information 2 [file peerj-14-20553-s002.pdf]

STROBE Statement—checklist of items that should be included in reports of observational studies

	Item No.	Recommendation	Page No.	Relevant text from manuscript
**Title and abstract**	1	(*a*) Indicate the study's design with a commonly used term in the title or the abstract	1	Precision Mapping of Mandibular Canal Dimensions: CBCT Insights
		(*b*) Provide in the abstract an informative and balanced summary of what was done and what was found	2	This retrospective split-mouth study analyzed 126 CBCT scans of 63 males and 63 females between the age group of 18 to 65 years from the faculty of Dentistry, Taibah University, Saudi Arabia. Measurements were BCT at nine points between the first premolar and third molar area, IAC diameter, nearest point of IAC to the mandible's lower border, MF position in relation to premolar apices and mandibular border, and the position of buccal shifting of the IAC. BCT showed a progressive increase from anterior to posterior regions in both genders, with the thickest measurements at the second and third molar regions. The MF position showed considerable individual variation, with 10% of female subjects exhibiting a

				coronal position relative to premolar apices. The IAC diameter ranged from 2.43 to 3.80 mm. The closest position of the IAC to the lower border was mostly in the second molar area, with the shortest distances being 2.50-3.40 mm. The canal mainly has a buccal shift in the second molar in 60-81.8% of the subjects.
**Introduction**				
Background/rationale	2	Explain the scientific background and rationale for the investigation being reported	1-2	The IAC shows various anatomical variations, increasing the risk of neurovascular complications during surgical procedures, ranging from paresthesia to complete anesthesia of the lower lip and chin (Naitoh et al. 2009) (Bartling et al. 1999). During third molar extractions, IAN injury rates range from 0.4% to 8.4%.(Jerjes et al. 2010) Despite the clinical significance, studies for comprehensive IAC mapping are limited especially in Middle Eastern populations. Existing researches often focus on specific anatomical features or regions, lacking detailed,

				comprehensive mapping and interpretation
Objectives	3	State specific objectives, including any prespecified hypotheses	2	This study aimed at a comprehensive mapping of the IAC using CBCT to assess gender and side-based mandibular anatomical variations, having direct clinical implications to various dental and maxillofacial procedures, to enhanced preoperative planning and reduced neurovascular complications risk
**Methods**				
Study design	4	Present key elements of study design early in the paper	3	This split-mouth study was conducted retrospectively for mapping the IAC of the mandible using CBCT scans from the College of Dentistry, Taibah University, KSA. Ethical approval was obtained from the ethics committee
Setting	5	Describe the setting, locations, and relevant dates, including periods of recruitment, exposure, follow-up, and data collection	3	This split-mouth study was conducted retrospectively for mapping the IAC of the mandible using CBCT scans from the College of Dentistry, Taibah University, KSA.<br>A total 126 CBCT scans from 63 male and 63 female subjects were involved in the study, with

				an age range of 18 to 65 years. The used sample size formula was n = ((Zα/2 · σ) / E) ². Where Zα/2 is the critical value for 95% confidence, σ is the standard deviation, and E is the margin of error. The minimum sample size was 35 per group, increased to 63 to improve statistical power.
Participants	6	(*Cross-sectional study*—Give the eligibility criteria, and the sources and methods of selection of participants	3	Inclusion criteria consisted of CBCT scans of adult patients (≥18 years) with fully erupted mandibular posterior dentition. Exclusion criteria included scans with significant artifacts, history of mandibular trauma or surgery, presence of mandibular pathological lesions and/or developmental anomalies
Variables	7	Clearly define all outcomes, exposures, predictors, potential confounders, and effect modifiers. Give diagnostic criteria, if applicable	3-4	*1. Buccal cortex thickness (BCT):*<br>*2. Buccal shifting location:*<br>*3. Location of the mental foramen (MF):*<br>*4. Diameter of the inferior alveolar canal:*<br>*5. Closest point from the inferior alveolar canal to the lower border of the mandible:*

Data sources/ measurement	8*	For each variable of interest, give sources of data and details of methods of assessment (measurement). Describe comparability of assessment methods if there is more than one group	*3-4*	*Measurement parameters:*<br>*1. Buccal cortex thickness (BCT):*<br>*BCT was measured at nine sites from the first premolar (tooth #4) to the third molar (tooth #8), including interdental regions, using CBCT cross-sectional views from the buccal cortex of the IAC to the most buccal point of the mandibular buccal cortex at the specified locations as shown in fig. 1.*<br>*2. Buccal shifting location:*<br>*The point of buccal shifting of the IAC was identified using panoramic, then monitored in axial CBCT views until the buccal shift was clearly observed and recorded relative to adjacent teeth.*<br>*3. Location of the mental foramen (MF):*<br>*The location of the MF was identified and measured from MF center to three different points in millimeters using the sagittal view of CBCT: the apices of the first premolar and second premolar, and the lower border of the mandible.*<br>*4. Diameter of the inferior*

				alveolar canal:<br>*The diameter of the IAC was measured at five locations next to the apices of first and second premolars and the first, second, and third molar teeth using the cross-sectional view from CBCT, then refined on the sagittal view to ensure accurate determination of the canal diameter as shown in fig. 2.*<br>*5. Closest point from the inferior alveolar canal to the lower border of the mandible:*<br>*Using the sagittal view of CBCT, the distance between the lower borders of the IAC and the mandible was measured at multiple points along the canal. The minimum distance was recorded, and its location was noted in relation to the corresponding tooth.*
Bias	9	Describe any efforts to address potential sources of bias	3	Image acquisition and analysis: All CBCT scans were analyzed using Carestream 3D Imaging Software (Carestream Dental LLC, Atlanta, USA). Patients were positioned in the Frankfort Horizontal plane prior to imaging. Scans were acquired at 90 kVp, 14.55 mAs, 160 µm

				voxel size, and 15.5-second. Images were reconstructed at 0.1 mm slice thickness and measurements were taken to 0.01 mm precision using digital calipers. All measurements were performed by two calibrated examiners. To ensure reliability, 20% of the scans were re-measured after two weeks to calculate intra- and inter-examiner reliability.
Study size	10	Explain how the study size was arrived at	3	A total 126 CBCT scans from 63 male and 63 female subjects were involved in the study, with an age range of 18 to 65 years. The used sample size formula was $n = ((Z\alpha/2 \cdot \sigma) / E)^2$. Where $Z\alpha/2$ is the critical value for 95% confidence, σ is the standard deviation, and E is the margin of error. The minimum sample size was 35 per group, increased to 63 to improve statistical power.

Continued on next page

Quantitative variables	11	Explain how quantitative variables were handled in the analyses. If applicable, describe which groupings were chosen and why		
Statistical methods	12	(*a*) Describe all statistical methods, including those used to control for confounding	4	Data was analyzed using IBM SPSS Statistics v20.0 (Armonk, NY: IBM Corp). Categorical data were expressed as numbers and percentages; comparisons used the Chi-square test, with Fisher's Exact or Monte Carlo corrections when >20% of cells had expected counts <5. Normality of continuous data was assessed using the Shapiro-Wilk test. Quantitative data were summarized as range, mean, and SD. Student's t-test was used for comparing two independent groups, and the paired t-test for related samples. Significance was set at $p \leq 0.05$.
		(*b*) Describe any methods used to examine subgroups and interactions		
		(*c*) Explain how missing data were addressed		
		Cross-sectional study—If applicable, describe analytical methods taking account of sampling strategy	3	A total 126 CBCT scans from 63 male and 63 female subjects were involved in the study, with an age range of 18 to 65 years. The used sample size formula was $n = ((Z\alpha/2 \cdot \sigma) / E)^2$. Where $Z\alpha/2$ is the critical value for 95% confidence, σ is the standard deviation, and E is the margin of error. The minimum sample size was 35 per group,

				increased to 63 to improve statistical power.
		(*e*) Describe any sensitivity analyses		
**Results**				
Participants	13*	(a) Report numbers of individuals at each stage of study—eg numbers potentially eligible, examined for eligibility, confirmed eligible, included in the study, completing follow-up, and analysed	4	This retrospective study analyzed 126 CBCT scans to map the IAC, various anatomical landmarks were measured bilaterally including BCT, IAC diameter, proximity of the IAC to the mandibular border, MF position, and buccal shifting location of IAC.
		(b) Give reasons for non-participation at each stage		
		(c) Consider use of a flow diagram		
Descriptive data	14*	(a) Give characteristics of study participants (eg demographic, clinical, social) and information on exposures and potential confounders		
		(b) Indicate number of participants with missing data for each variable of interest		
		(c) *Cohort study*—Summarise follow-up time (eg, average and total amount)		
Outcome data	15*			
		Cross-sectional study—Report numbers of outcome events or summary measures	*1*	*BCT showed a progressive increase from anterior to posterior regions in both genders, with the thickest measurements at the second and third molar regions. The MF position showed considerable individual variation, with 10% of female subjects exhibiting a coronal position relative to premolar apices. The IAC diameter ranged from 2.43 to 3.80 mm. The closest*

				position of the IAC to the lower border was mostly in the second molar area, with the shortest distances being 2.50-3.40 mm. The canal mainly has a buccal shift in the second molar in 60-81.8% of the subjects.
Main results	16	(*a*) Give unadjusted estimates and, if applicable, confounder-adjusted estimates and their precision (eg, 95% confidence interval). Make clear which confounders were adjusted for and why they were included	4	Buccal Cortex Thickness:<br>The BCT was measured at nine locations bilaterally, from the first premolar to the third molar region with no statistically significant differences between male and female or right and left sides ($p > 0.05$). However, notable anatomical patterns were identified.<br>For female subjects, the BCT was generally thicker than in male subjects, with a maximum difference of 0.92 and 0.89 mm on the left side and the right side respectively. The maximum recorded value for BCT in females was at tooth 47 by 6.05 ± 1.34 mm, while in males was measured at the midpoint between teeth 37 and 38 by 5.14 ± 1.24 mm and at the midpoint between teeth 47 and 48 by 5.42 ± 1.58 mm as shown in fig. 3.<br>At the premolar region (tooth 34), the mean BCT was 2.27 ± 0.95 mm

in males and 2.45 ± 1.20 mm in females. Measurements at this location were only possible in 27.27% of males and 20% of females, representing the anatomical looping of the nerve at the left side. Similarly, at tooth 44, measurements were possible in 18% of males and 40% of females as shown in fig. 3.

Table 1 showed the detailed measurements of BCT at all locations for both genders and sides. The data shows a consistent pattern of gradual increase in thickness from the premolar to the molar region, with the thickest measurements recorded in the second and third molar regions.

Mental Foramen Location:

The position of the MF was assessed in relation to the apices of the first and second premolars and the lower border of the mandible. No statistically significant differences were found between sides or genders ($p > 0.05$) as shown in table 1.

In male subjects, the mean distance from the MF to the lower border of the mandible was 11.51 ± 1.83 mm on the left side and 10.71 ± 1.99

mm on the right side. The mean distances from the MF to the apices of the first and second premolars on the left side were 7.78 ± 2.40 mm and 6.64 ± 2.45 mm, respectively. On the right side, these distances were 7.98 ± 2.22 mm and 7.51 ± 2.70 mm, respectively.

In female subjects, the mean distance from the MF to the lower border of the mandible was 10.05 ± 1.62 mm on the left side and 10.30 ± 1.86 mm on the right side. The mean distances from the MF to the apices of the first and second premolars were 7.42 ± 2.27 mm and 6.11 ± 3.30 mm on the left side, and 5.98 ± 3.94 mm and 6.03 ± 4.25 mm on the right side.

An important observation was the presence of negative values in 10% of female subjects bilaterally, indicating a coronal position of the MF relative to the premolar apices. This anatomical variation was not observed in male subjects.

Diameter of the Inferior Alveolar Canal:

The diameter of the IAC was measured at five locations corresponding to teeth #4 through #8. No significant differences were

found between genders or sides ($p > 0.05$) as shown in table 2.

In male subjects, the canal diameter ranged from 2.43 ± 1.82 mm (at tooth 34) to 3.80 ± 0.73 mm (at tooth 38) on the left side, and from 2.47 ± 0.38 mm (at tooth 44) to 3.71 ± 0.78 mm (at tooth 45) on the right side.

In female subjects, the canal diameter ranged from 2.60 ± 0.71 mm (at tooth 34) to 3.41 ± 0.66 mm (at tooth 35) on the left side, and from 2.45 ± 0.71 mm (at tooth 44) to 3.78 ± 1.74 mm (at tooth 45) on the right side.

Variability in IAC diameter was assessed by coefficient of variation (CV), it was highest in premolar regions. In males, CV ranged from 12.6% (tooth 47) to 35.3% (tooth 35). In females, it ranged from 14.2% (tooth 38) to 46% (tooth 45), indicating significant anatomical variation.

Closest Point of Inferior Alveolar Canal to the Lower Border of the Mandible:

The minimum distance between the IAC and the lower border of the mandible was measured. Although not statistically significant, the

canal was generally closer to the lower border in males than in females, and on the right side compared to the left side.

In male subjects, the minimum distances were 4.56 ± 1.50 mm on the left side at teeth 36 and 37 and 4.85 ± 1.68 mm on the right side at tooth 47.

In female subjects, the minimum distances were 4.96 ± 1.08 mm on the left side at tooth 37 and 5.09 ± 0.96 mm on the right side at tooth 48.

The IAC was closest to the lower border most frequently in the second molar region. In females, this occurred at tooth 47 (60%) and 37 (40%) bilaterally. Among males, the right side showed the highest frequency (72.7%), followed by the left (45.5%).

Location of the Buccal Shifting of Inferior Alveolar Canal:

Buccal shifting of the IAC was most frequent at the first and second molar regions in both genders. In males, it occurred at second molar tooth by 81.8% and 72.7% of cases on the right and the left sides respectively. In females, it was seen at the second molar in

		70% of cases on the right and 60% on the left.
	(*b*) Report category boundaries when continuous variables were categorized	
	(*c*) If relevant, consider translating estimates of relative risk into absolute risk for a meaningful time period	

Continued on next page

Other analyses	17	Report other analyses done—eg analyses of subgroups and interactions, and sensitivity analyses		
**Discussion**				
Key results	18	Summarise key results with reference to study objectives	5-7	Buccal Cortex Thickness:<br>A progressive increase in BCT from premolar to molar was noted<br>Mental Foramen Position:<br>MF position showed considerable variation in its position relative to premolar apices.<br>Diameter of the Inferior Alveolar Canal:<br>The IAC diameter varied significantly across different regions and individuals, with coefficients of variation ranging from 12.6% to 46%, underscoring the need for patient-specfic assessment before invasive procedures.<br>Proximity of the Inferior Alveolar Canal to the Inferior Border of the Mandible:<br>IAC was typically closer to the mandibular lower border in males on the right side, though these differences were not statistically significant.<br>Buccal displacement: increases IAN injury risk due to greater vulnerability during buccal access
Limitations	19	Discuss limitations of the study, taking into account sources of potential bias or imprecision. Discuss	7-8	Limitations and Future Directions:

		both direction and magnitude of any potential bias		This study has several limitations. First, the sample was limited to a single geographical area (Saudi Arabia). Second, certain anatomical measurements, such as bifid canals, accessory mental foramina, and canal-to-root proximity, were not taken and should be included in future studies for more complete anatomical mapping. Finally, the creation of artificial intelligence algorithms for automatic detection and measurement of anatomical landmarks has the potential to improve the efficiency and accuracy of preoperative evaluations, a promising avenue for future research investigations.
Interpretation	20	Give a cautious overall interpretation of results considering objectives, limitations, multiplicity of analyses, results from similar studies, and other relevant evidence		
Generalisability	21	Discuss the generalisability (external validity) of the study results		
**Other information**				
Funding	22	Give the source of funding and the role of the funders for the present study and, if applicable, for the original study on which the present article is based	Not applicable	No fund

*Give information separately for cases and controls in case-control studies and, if applicable, for exposed and unexposed groups in cohort and cross-sectional studies.

Note: An Explanation and Elaboration article discusses each checklist item and gives methodological background and published examples of transparent reporting. The STROBE checklist is best used in conjunction with this article (freely available on the Web sites of PLoS Medicine at http://www.plosmedicine.org/, Annals of Internal Medicine at http://www.annals.org/, and Epidemiology at http://www.epidem.com/). Information on the STROBE Initiative is available at www.strobe-statement.org.
